# Physical impairments in individuals with Long COVID

**DOI:** 10.3389/fspor.2025.1511942

**Published:** 2025-01-30

**Authors:** Imane Salmam, Kadija Perreault, Krista L. Best, Imane Zahouani, Gilles Drouin, Jean Tittley, François Desmeules, Alexandre Campeau-Lecours, Simon Beaulieu-Bonneau, Jean-Sébastien Paquette, Simon Deslauriers, Sarah-Maude Brouillard, Katherine Lepage, Jean-Sébastien Roy

**Affiliations:** ^1^ Centre for Interdisciplinary Research in Réactualisation and Social Integration, CIUSSS de la Capitale Nationale, Quebec City, QC, Canada; ^2^ School of Rehabilitation Sciences, Faculty of Medicine, Université Laval, Quebec City, QC, Canada; ^3^ Hospital Maisonneuve-Rosemont Research Center, Montreal, QC, Canada; ^4^ School of Rehabilitation, Faculty of Medicine, Université de Montréal, Montreal, QC, Canada; ^5^ Department of Mechanical Engineering, Faculty of Science and Engineering, Université Laval, Quebec City, QC, Canada; ^6^ School of Psychology, Faculty of Social Sciences, Université Laval, Quebec City, QC, Canada; ^7^ VITAM—Research Centre on Sustainable Health, Quebec City, QC, Canada; ^8^ Département de Médecine Familiale et de Médecine d’urgence, Faculté de Médecine, Université Laval, Quebec City, QC, Canada; ^9^ Laboratoire ARIMED, Groupe de Médecine de Famille du Nord de Lanaudière, CISSS Lanaudière, Quebec City, QC, Canada; ^10^ Patient Partners, Quebec City, QC, Canada

**Keywords:** Long COVID, physical impairments, persistent symptoms, post-COVID-19 syndrome, fatigue

## Abstract

**Objective:**

The primary objective was to compare the physical capacities of individuals with Long COVID [Long COVID group (LCG)] to those who had COVID-19 but did not develop persistent symptoms [short COVID group (SCG)], and to individuals without a history of COVID-19 [control group (CG)]. The secondary objectives were to provide a comprehensive profile of sociodemographic and COVID-19 history of individuals with Long COVID, considering factors such as sex, gender, hospitalization, time since onset, and comorbidities, and b) identify self-reported and objective clinical measures explaining health-related quality of life (HRQoL) in individuals with Long COVID.

**Methods:**

A total of 120 adults were included in each of the groups. Participants completed self-reported assessments covering HRQoL, comorbidities, pain, sleep, and fatigue. Physical assessments included handgrip strength (HGS), Short Physical Performance Battery (SPPB), 6-minute-walk-test (6MWT), perceived exertion during the 6MWT (Modified-Borg Scale), and daily step count during a 7-day period.

**Results:**

Mean age (mean [SD]) for LCG, SCG, and CG was 44.2 [11.2], 42.1 [16.4], and 46[15.9], respectively. LCG showed significantly higher pain, comorbidities, and fatigue, along with lower HRQoL and sleep quality compared to the other groups. HGS, SPPB, and 6MWT performance were also significantly lower in LCG, while perceived exertion during 6MWT was higher. Finally, the number of steps per day was significantly lower in LCG. Higher prevalence of obesity and comorbidities were identified among those hospitalized after COVID-19. Fatigue, pain, comorbidities, and Step count were the variables explaining HRQoL for LCG (*R*2: 0.58; *F* = 35.9).

**Conclusion:**

Long COVID individuals, on average 329 [146] days post-infection, experience enduring physical and health-related challenges, with significant implications for their overall well-being.

## Introduction

Since December 2019, the novel coronavirus known as severe acute respiratory syndrome coronavirus 2 (SARS-CoV-2 or COVID-19), has spread worldwide, resulting in an estimated 6.9 million deaths (1, 2). Additionally, 10%–30% of COVID-19 survivors experience persistent symptoms (3, 4), a condition referred to as “Long COVID syndrome” or “post-acute sequelae of SARS-CoV-2 infection”. It entails the persistence or emergence of new symptoms three months after the initial infection (5). These symptoms encompass fatigue, dyspnea, neurological symptoms, and chest pain, among others (6, 7). So far, studies suggest that being female (8), having comorbidities (3), and being obese (9) are associated with a higher risk of developing Long COVID.

The severity and duration of Long COVID symptoms vary considerably among individuals (10). Consequently, the impact on quality of life varies depending on the extent of these symptoms, potentially leading to substantial limitations in social and physical activities (11, 12). For instance, data from 120 patients uncovered enduring symptoms, such as fatigue (55%), dyspnea (42%), memory difficulties (34%), concentration difficulties (28%), and sleep disorders (31%) persisting even three months post-discharge from hospital (13). Furthermore, individuals with Long COVID often report worsening symptoms with physical exertion, leading to exercise intolerance (14). This condition, characterized by a reduced capacity for physical activity that is exacerbated even by mild exertion (15), has become a prominent feature of Long COVID (16). Physiologically, exercise intolerance in Long COVID may stem from dysregulated autonomic function, impaired oxygen uptake, or persistent inflammation, further limiting physical capacity (17). This is supported by evidence of significant reductions in exercise capacity, as measured by peak oxygen uptake (VO_2peak_) and performance in the 6-minute walk test (6MWT), among Long COVID patients compared to healthy controls (18, 19). Other findings from an online survey involving 3,762 participants highlighted lingering symptoms and occurrences of relapses associated with exercise, physical or mental activity, and stress seven months post-infection (20). Despite growing research, our understanding of Long COVID remains limited. Most studies rely either on retrospective data or online questionnaire, often lacking comparative analyses with individuals who do not experience symptoms or were never infected with COVID-19. A study integrating self-reported questionnaires, and physical evaluations could provide valuable insights into Long COVID. Furthermore, the lack of categorization based on risk hinders a comprehensive understanding of the impacts of Long COVID, thereby limiting the development of more effective treatment strategies.

To address these knowledge gaps, this study's primary objective is to compare the physical capacities of individuals with Long COVID with those of individuals who contracted COVID but did not develop persistent symptoms, and individuals without a history of COVID infection. The secondary objectives are: (1) to provide a comprehensive sociodemographic and COVID history profile of individuals with Long COVID, considering factors such as sex, gender, hospitalization, time since onset, and the number of comorbidities; and (2) to determine which self-reported and clinical measures can explain the quality of life in individuals with Long COVID.

## Methods

### Participants

Three groups of age- and sex-matched adults were recruited: Long COVID group (LCG) comprised individuals experiencing persistent symptoms for more than 12 weeks; Short COVID group (SCG) included individuals who had developed COVID-19 but recovered without persistent symptom; Control group (CG) consisted of individuals who did not develop COVID-19.

Individuals in the COVID groups had tested positive for COVID-19 through PCR or rapid antigen testing and were recruited no earlier than 12 weeks after infection. Participants in the LCG self-reported at least one symptom persisting for at least 12 weeks following their initial COVID-19 infection, aligning with the WHO definition (5). Participants in the SCG did not experience any lingering symptoms beyond 4 weeks from their initial COVID-19 infection. Participants of the CG were individuals without any known history of COVID-19 infection. Recruitment took place between June 2021 and March 2023. Ethical approval was granted by the *Comité d'éthique de la recherche du CIUSSS de la Capitale-Nationale* (2022-2328) and the *Comité d'éthique de la recherche du CIUSSS de l'Est-de-l'Île-de-Montréal* (2022-2811). The study protocol was published and more details on the study design and on the selected variable and their psychometric properties can be found in the protocol (21).

### Study design

This study employed a cross-sectional design and adhered to the STROBE (Strengthening the Reporting of Observational Studies in Epidemiology) guidelines for cross-sectional studies to ensure thorough and transparent reporting (22). Participants were recruited from various sources, including medical clinics in Quebec City and Montreal (e.g., family medicine groups), electronic mailing lists targeting students and current or former employees of Université Laval, social media platforms such as Facebook, and advertisements on a website for retirees and seniors. During a telephone screening, the experimental procedures and tests were thoroughly explained to all interested individuals to ensure they fully understood the study requirements before providing consent. This process ensured that participants were well-informed and had the physical and mental capacity to complete the evaluations safely and comfortably, with their individual limitations respected throughout process. Afterward, participants signed an online consent form and completed web-based self-reported questionnaires (hosted on the REDCap platform) (23). These questionnaires assessed health-related quality of life, comorbidity, sleep quality, pain and pain-related disabilities, and fatigue. They were selected to address the most commonly reported physical symptoms among individuals with Long COVID (23).

Within one week of inclusion, participants attended an in-lab evaluation either in Quebec City or in Montreal. This session involved answering questions on sociodemographic, medication usage, and vaccination status, along with measurements of weight and height. Participants in the COVID groups provided details on their COVID-19 history and symptoms. Then participants completed objective clinical tests to assess physical function (grip strength, balance, gait speed, gait endurance). All clinical tests were performed in a uniform sequence, from the least to the most strenuous. Breaks were provided as necessary (24–27). Finally, participants were instructed to wear a fitness tracker watch for seven days in their real-life environments. Evaluators were aware of participants' groups to accommodate potential fatigue in individuals with Long COVID.

### Self-reported questionnaires

#### Health-related quality of life

The EQ-5D-5L assesses health-related quality of life (HRQoL) across five dimensions (mobility, self-care, usual activities, pain/discomfort, anxiety/depression) using a five-point scale (28).

#### Comorbidities

The Self-Administered Comorbidity Questionnaire (SCQ) is designed to capture information on 12 common medical conditions (29). Participants indicate whether they have any of these medical conditions, whether they are receiving treatment for them, and if these conditions resulted in activity limitations. The maximum global score a participant can achieve is 45 points.

#### Sleep quality

The Pittsburgh Sleep Quality Index (PSQI) is designed to assess the quality and patterns of sleep over the past month (30). A 4-point Likert is used to asses seven distinct aspects of sleep. The cumulative score ranges from 0 to 21. The higher the score, the worse the sleep quality.

#### Pain and pain-related disabilities

The Brief Pain Inventory Short Form (BPI-SF) is an 11-item questionnaire specifically developed to assess both the intensity of pain and its impact on a person's daily functioning (31). It includes 4 questions about pain intensity (Pain Severity subscale), and 7 questions about pain interference (Pain Interference subscale). Higher scores indicate more severe pain.

#### Fatigue

The Fatigue Severity Scale (FSS) is designed to assess the severity of fatigue and its impact on an individual's daily activities and lifestyle (32). The scale consists of nine items, each rated on a seven-point scale. Final score was calculated as the sum of all the items. A higher score indicates more pronounced fatigue and a greater impact on daily activities.

### Objective clinical tests

#### Hand grip strength

In a seated position, hand grip strength (HGS) for both hands was assessed (90° of elbow flexion, wrist neutral) using a handgrip dynamometer (Jamar, JA Preston Corporation, New York, USA) (26). Each participant performed three trials, and the highest achieved strength (in kilograms) was utilized for subsequent analysis.

#### Short physical performance battery (SPPB)

The SPPB assesses lower extremity function when completing three tasks that simulate everyday activities (27): static standing balance, gait speed, and chair transfers. Each task is rated on a scale ranging from 0 (inability) to 4 (highest level of ability). A cumulative final score is summed (range of 0–12).

#### 6-minute walk test (6MWT)

The 6MWT is a submaximal evaluation of endurance and functional capacity. It entails measuring the maximum distance an individual can cover within a span of 6 min (25). The distance covered during the 6-minute period is measured using a measuring wheel.

#### Perceived exertion level

Perceived Exertion Level [0 (no exertion at all) to 10 (maximal exertion)] was collected using the Modified Borg Scale (33) at each minute during the 6MWT.

#### Physical activity

Following the in-lab evaluation, participants were instructed to wear a fitness tracker watch (Garmin Forerunner 35) continuously for a span of seven consecutive days. Data were then manually retrieved on-site from the Garmin Connect platform. Daily averages for resting heart rate, number of steps, and intensive minutes were computed over the 7-day recording period.

### Sample size

As statistical analyses are mostly based on maximum-likelihood generalized ANOVA, the sample size should target around 60 participants per group (34). In this study, 120 participants/group is justified, as secondary analysis aims to provide a comprehensive sociodemographic and COVID history profile of individuals with Long COVID. This includes comparisons based on factors such as sex, gender, hospitalization, time since onset, and the number of comorbidities.

## Statistical analysis

For the primary objective of assessing differences between LCG and SCG and CG for self-reported and physical variables, one-way analysis of variance (ANOVA) was used when the difference between groups adhered to the assumptions for parametric analysis. Kruskal-Walli's test was used when the assumptions were not met. *post hoc* comparisons were performed using Tukey-B. Missing data in the study were addressed using mean imputation.

For secondary objectives: (1) a sociodemographic and COVID history profile for the Long COVID group was generated, categorizing information based on sex (men, women), gender (men, women, other), hospitalization status related to COVID-19 infection (hospitalized, non-hospitalized), time since infection (<6 months, between 6 and 12 months, >12 months) and number of comorbidities (0, 1 or 2, 3 or more). A comparison between subgroups in each categorization was conducted using a one-way ANOVA; (2) a backward stepwise linear regression was used with EQ-5D-5L scores as the dependent variable. Backward elimination involved starting with all candidate variables including sex, age, BMI, hospitalization, number of infections, time since infection, vaccinations, FSS, BPI-Severity, BPI-Interference, SCQ, PSQI, HGS, SPPB, 6MWT, average resting heart rate, and average step count per day. They were tested one by one for statistical significance, deleting any that were not significant. All data analysis was conducted using *R* software (version 4.3.1).

### Difference with the planned protocol

This article primarily focuses on physical function, enhancing our understanding of the profiles of individuals affected by long COVID. It categorizes participants based on gender, comorbidities, time since infection, and hospitalization, while cognitive variables are reserved for analysis in a separate publication. Given the numerous variables involved, we chose to split the longitudinal analyses to gain a clearer insight into the differences between groups and their associated factors. The upcoming longitudinal article will explore changes over time among the three groups and aim to identify key variables that predict quality of life at six months. Additionally, frailty assessment was also excluded, recognizing its subjectivity and potential inter-rater variation (21).

## Results

**Figure 1:**
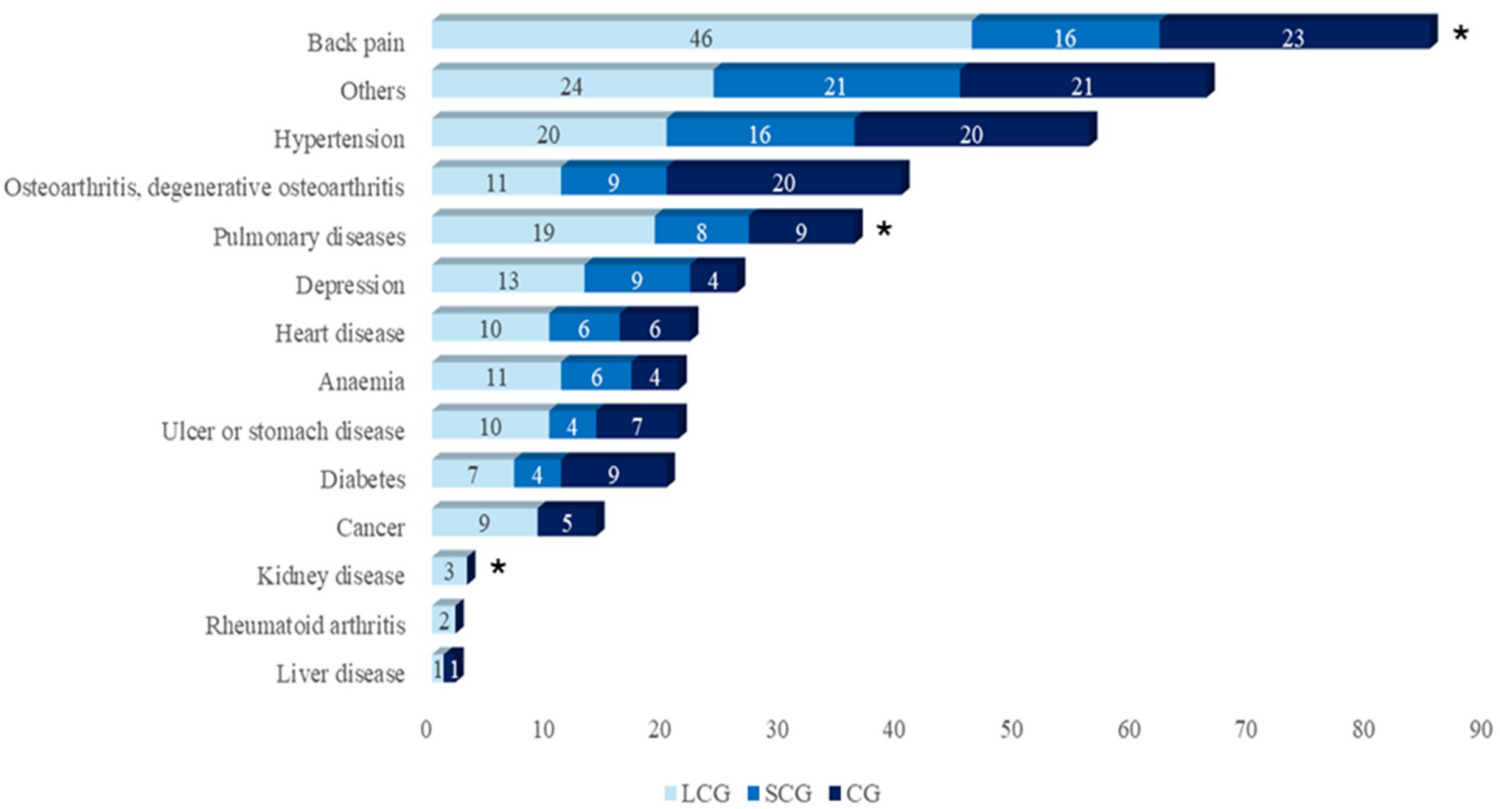
Distribution of comorbidities by group (*n* = 120 per group). LCG, Long COVID Group; SCG, Short COVID Group; CG, Control Group. * Statistically significant differences using Chi-squared test (*p* < 0.05).

A total of 360 adults participated in this study, distributed across three groups of 120 age- and sex-matched adults. Mean (SD) age was 44.2 (11.2), 42.1 (16.4) and 46.0 (15.9) years for LCG, SCG and CG, respectively. Mean (SD) time from infection in days was 329 (146) and 274 (218) in LCG and SCG, respectively. In the LCG, 110 participants (91.7%) reported having one or more comorbidity, compared to 62 (51.7%), and 68 (56.7%) in SCG and CG, respectively. Figure 1 shows distribution of comorbidities by group. As there was no difference between sex and self-identified gender within the sample, results are exclusively presented based on sex. Characteristics of all groups are summarized in Table 1. The top five symptoms persisting in the LCG at the time of assessment were fatigue (80%), difficulty concentrating (73%), shortness of breath (71%), memory difficulties (68%), and sleep disorders (57%) (Table 2).

**Table 1:** Participants' characteristics.

	LCG	SCG	CG	*P* value
	*n* = 120	*n* = 120	*n* = 120	
Sociodemographic				
Age, years; mean (SD)	44.2 (11.2)	42.1 (16.4)	46.0 (15.9)	0.12
Women; *n* (%)	102 (85)	90 (75)	90 (75)	0.095
Body Mass Index, kg/m^2^; mean (SD)	27.5 (6.2)	25.4 (5.1)	27.4 (6.8)	0.009
Smoking status; *n* (%)				0.026
Former smoker	26 (21.7)	23 (19.2)	25 (20.8)	
Never smoked	86 (71.7)	89 (74.2)	85 (70.8)	
Active smoker	6 (5.0)	8 (6.6.)	10 (8.4)	
Missing data	2 (1.6)	0 (0.0)	0 (0.0)	
Education; *n* (%)				0.13
No high school diploma	1 (0.8)	0 (0.0)	2 (1.7)	
High school diploma or equivalent (DES) and college certificate/diploma	31 (25.8)	30 (25.0)	29 (24.2)	
University diploma or degree below bachelor's or Trades certificates and diplomas	19 (15.8)	8 (6.7)	12 (10.0)	
Bachelor's degree	41 (34.2)	34 (28.3)	39 (32.5)	
Master's or Doctoral degree	28 (23.3)	48 (40.0)	38 (31.6)	
Ethnicity; *n* (%)				0.23
White	117 (97.5)	115 (95.8)	110 (91.7)	
Other	3 (2.5)	5 (4.2)	10 (8.3)	
COVID-19 History				
Time since first infection, days; mean (SD)	329 (146)	274 (218)	N/A	0.02
Hospitalization; *n* (%)	14 (11.7%)	2 (1.7%)	N/A	0.867
Length of hospitalization, days; mean (SD)	6.0 (5.5)	2.0 (1.3)	N/A	0.002
Number of COVID-19 infections; *n* (%)				0.16
1 infection	116 (96.7)	109 (90.8)	N/A	
2 infections	4 (3.3)	10 (8.5)		
3 infections	0	1 (0.85)		
Vaccination status; *n* (%)				<.001
Not vaccinated	5 (4.2)	2 (1.7)	6 (5.0)	
1 dose	24 (20.0)	1 (0.8)	2 (1.7)	
2 doses	67 (55.8)	29 (24.2)	20 (16.7)	
>3 doses	23 (19.2)	88 (73.3)	91 (75.8)	
Missing data	1 (0.8)	0 (0.0)	1 (0.8)	

LCG, Long COVID Group; SCG, Short COVID Group, CG, Control group.

*P*-values for continuous variables were calculated using one-way ANOVA, while *p*-values for categorical variables were derived from a chi-squared test.

**Table 2:** List of symptoms experienced by participants with long COVID (*n* = 120).

Symptoms	Symptom experienced within the first 3 months after the infection; *n* (%)	Symptoms experienced during the study period; *n* (%)
Fatigue	114 (95)	96 (80)
Difficulty concentrating	103 (86)	88 (73)
Shortness of breath	98 (82)	85 (71)
Memory difficulties	94 (78)	81 (68)
Sleep disorders	83 (69)	68 (57)
Joint pain	82 (68)	67 (56)
Headache	91 (76)	65 (54)
Muscle weakness	79 (66)	60 (50)
Dizziness	75 (63)	55 (46)
Feeling of pressure	70 (58)	51 (43)
Sense of smell	87 (73)	45 (38)
Anxiety	49 (41)	45 (38)
Heart problems	50 (42)	40 (33)
Visual problems	42 (35)	32 (27)
Circulatory problems	40 (33)	31 (26)
Hair loss	40 (33)	29 (24)
Loss of balance	40 (33)	28 (23)
Digestive disorders	41 (34)	26 (22)
Tinnitus	32 (27)	25 (21)
Involuntary contractions	27 (23)	18 (15)
Skin problems	26 (22)	17 (14)
Diarrhea	40 (33)	14 (12)
Vocal problems	18 (15)	14 (12)
Swallowing problems	14 (12)	11 (9)
Toothache	15 (13)	11 (9)
Constipation	16 (13)	10 (8)
Kidney problems	8 (7)	3 (3)

Statistically significant differences were identified between participants in LCG and both SCG and CG for all questionnaires (*p* < .001) and physical tests (*p* ≤ .001). Participants in the LCG had lower HRQoL, higher comorbidity score, greater pain and pain-related disabilities, lower sleep quality, and higher fatigue level. Moreover, participants in the LCG had lower HGS, SPPB score, walking distance during the 6MWT, and higher perceived exertion during the 6MWT. Finally, participants in the LCG had higher resting heart rate (*p* = 0.002), and lower step count per day (*p* < .001) compared to SCG and CG. Detailed results are presented in Table 3.

**Table 3:** Self-reported questionnaires and physical evaluation.

	LCG (*n* = 120)	SCG (*n* = 120)	CG (*n* = 120)	*p* value	Mean difference (95% CI)			
					LCG vs. SCG	Cohen's d	LCG vs. CG	Cohen's d
Self-Reported Questionnaires; mean (SD)								
EQ-5D-5L	0.8 (0.1)	1.0 (0.03)	1.0 (0.1)	<.001*	−0.2 (−2.7 to −2.1)*	−2.4	−0.2 (−2.6 to −2.0)*	−2.3
PSQI	9.5 (4.5)	4.9 (2.7)	5.1 (3.1)	<.001*	4.7 (1.1–1.6)*	1.3	4.5 (1.0–1.5)*	1.3
BPI Pain Severity	3.2 (2.0)	0.9 (0.9)	1.0 (1.2)	<.001*	2.3 (1.3–1.9)*	1.6	2.1 (1.2–1.8)*	1.5
BPI Pain Interference	3.4 (2.5)	0.5 (0.8)	0.5 (0.9)	<.001*	3.0 (1.6–2.1)*	1.8	2.9 (1.5–2.1)*	1.8
SCQ	4.2 (3.7)	1.6 (2.0)	2.5 (2.9)	<.001*	2.6 (0.6–1.1)*	0.9	1.7 (0.3–0.8)*	0.6
FSS^a^	46.8 (11.9)	18.6 (9.3)	19.2 (10.7)	<.001*	28.2 (2.3–2.95)*	2.6	27.6 (2.25–2.9)*	2.6
Objective Clinical Tests; mean (SD)								
HGS	31.5 (9.9)	36.0 (10.7)	35.5 (9.9)	0.001*	−4.5 (−0.7 to −0.2)*	−0.4	−4.0 (−0.6 to −0.1)*	−0.4
SPPB	10.6 (1.9)	11.8 (0.6)	11.7 (0.8)	<.001*	−1.3 (−1.3 to −0.8)*	−1.0	−1.2 (−1.2 to −0.7)*	−0.95
6MWT	464.4 (131)	603.0 (73.3)	567.9 (87.9)	<.001*	−138.6 (−1.7 to −1.1)*	−1.4	−103.4 (−1.3 to −0.8)*	−1.0
Highest perceived exertion during 6MWT	3.9 (1.7)	2.4 (1.3)	2.40 (1.6)	<.001*	1.5 (0.7–1.2)*	0.9	1.5 (0.7–1.2)*	0.9
Physical Activity (Fitness Tracker Watch); mean (SD)^b^								
	*N* = 105	*N* = 96	*N* = 98					
Highest HR	137.8 (19.8)	138.5 (12.8)	138.8 (12.4)	0.9	−0.7 (−0.3 to 0.2)	−0.05	−1.0 (−0.3 to 0.2)	−0.1
HR at rest	66.5 (6.4)	62.9 (7.0)	64.2 (8.0)	0.002*	3.6 (0.2–0.8)*	0.5	2.3 (0.05–0.6)	0.3
Intensive minutes	18.2 (19.3)	13.4 (19.1)	20.1 (26.3)	0.09	4.8 (−0.1 to 0.5)	0.2	−1.8 (−0.4 to 0.2)	−0.08
Step count/day	5,409 (2,388)	7,993 (3,068)	7,490 (3,789)	<.001*	−2,584 (−1.1 to −0.5)*	−0.8	−2,081 (−0.9 to −0.4)*	−0.7

LCG, Long COVID Group; SCG, Short COVID Group; CG, Control group; SE, Standard error; PSQI, Pittsburgh Sleep Quality Index; EQ-5D-5L, EuroQol Group's 5-Dimension 5-Level Questionnaire; SCQ, Self-Administered Comorbidity Questionnaire; BPI-SF, Brief Pain Inventory—Short Form; FSS, Fatigue Severity Scale; HGS, Hand grip strength; SPPB, Short Physical Performance Battery; 6MWT, 6 min walk test; HR, heart rate.

* Significant difference (*p* < 0.05).

^a^ Please note that the FSS score consists of 8 items instead of 9 due to an error during data collection, resulting in the deletion of the last FSS item.

^b^ Data regarding physical activity was gathered from all participants. Missing data are attributed to malfunctions with the watches.

Regarding comparisons based on categorized variables (sex, hospitalization status, time since infection, and number of comorbidities) within the Long COVID group, no statistically significant differences were found between men (*n* = 18) and women (*n* = 102) on all questionnaires (*p* > 0.05). However, men had significantly higher HGS (*p* < .001) and covered a longer distance during 6MWT (*p* = 0.04). When categorized by hospitalization status, a significant difference was observed in the SCQ questionnaire (*p* = 0.02) with hospitalized participants (*n* = 14) scoring higher, indicating a greater burden of comorbidities. Similarly, when categorized by the number of comorbidities, significant differences were identified for EQ-5D-5L (*p* < .001), BPI-Severity (*p* = 0.002), and BPI-Interference (*p* = 0.02) indicating better HRQoL and less pain and pain-related disability in those with fewer comorbidities. Additionally, the 6MWT showed significant differences (*p* = 0.049), with those having more comorbidities covering shorter distances. When categorized by the time since infection, a significant difference in FSS scores was identified (*p* = 0.03), with higher fatigue levels among participants whose infection occurred less than six months prior. However, no significant differences in objective physical tests were found based on time since infection. Detailed results are provided in Supplementary Table S1.

The most influential independent variables explaining HRQoL in individuals with Long COVID were FSS, BPI-Interference, SCQ, and step count. The overall model demonstrates a strong fit to the data (Adjusted *R*^2^: 0.58; *F* = 35.9), indicating that our model accounts for 58% of the observed variance in the EQ-5D-5L. Detailed results are presented in Table 4.

**Table 4:** Linear regressions.

*R*	Adjusted *R*^2^	*F*	*p* value
0.771	0.578	35.9	<.001

**Table:** 

Model coefficients—EQ-5D-5L							
95% Confidence interval							
Predictor	Estimate	SE	Lower	Upper	*t*	*p*	Stand. estimate
Intercept	0.93063	0.04535	0.84063	1.02062	20.52	<.001	
SCQ	−0.00641	0.00226	−0.01089	−0.00192	−2.83	0.006	−0.204
BPI Pain Interference	−0.01469	0.00370	−0.02202	−0.00735	−3.97	<.001	−0.309
FSS	−0.00315	7.73 × 10^−4^	−0.00468	−0.00161	−4.07	<.001	−0.313
Step count	1.48 × 10^−5^	3.54 × 10^−6^	7.78 × 10^−6^	2.18 × 10^−5^	4.18	<.001	0.299

EQ-5D-5L, EuroQol Group's 5-Dimension 5-Level Questionnaire; SCQ, Self-Administered Comorbidity Questionnaire; BPI, Brief Pain Inventory; FSS, Fatigue Severity Scale; SE, Standard error of the estimate; Stand. Estimate, Standardized estimate.

## Discussion

Our study reveals that even on average 11 months post-infection, individuals with Long COVID show higher levels of disabilities and lower physical capacities compared to those who did not experience persistent symptoms after their COVID-19 infection and those without a history of COVID-19.

These impairments in physical capacities may stem from underlying physiological and muscular changes associated with Long COVID. Emerging evidence suggests that persistent symptoms like fatigue and muscle weakness could be linked to muscular dysfunctions at a cellular level, affecting overall physical performance (35). The lower physical capacity among participants in the LCG may be attributed to the presence of myopathy and skeletal muscle issues (36). Muscle biopsies from individuals with Long COVID confirmed the existence of myopathy, providing support for the association between myopathy and fatigue in Long COVID (36). In line with these findings, a previous study indicated that 50% of individuals with Long COVID exhibited muscle weakness (34). Additionally, all participants from that study displayed histological changes, including 38% with muscle fiber atrophy and 56% showing signs of fiber regeneration (34). These results suggest that histological changes, encompassing mitochondrial alterations, inflammation, and capillary injury observed in muscle biopsies, may contribute to fatigue, partly due to a diminished energy supply (34). This, in turn, hinders muscle function, strength, and endurance, directly impacting an individual's performance in physical tests.

The relationship between Long COVID and physical capacity remains complex, with the factors explaining functional capacities still to be clarified, as findings across studies have been inconsistent. Berg et al. reported no significant differences in maximal oxygen uptake or pulmonary function but observed reduced 6MWT performance, likely due to fatigue and deconditioning, with participants exceeding pre-pandemic predicted values (37). In contrast, our larger cohort of 120 participants showed greater reductions, with an average 6MWT distance of 464 (131) meters compared to 606 (118) meters in Berg et al.'s study, indicating greater physical limitations (37). Similar to our results, several studies have reported lower 6MWT performance (38–41). For instance, a cross-sectional study of 34 Long COVID individuals found significantly reduced 6MWT distance of 423 (7) meters, with a mean difference of 94 (19) meters compared to healthy controls (42). The authors suggested that reduced performance could be linked to intrapulmonary or extrapulmonary causes (42). Supporting the latter, Mooren et al. reported significantly reduced physical fitness, including lower peak oxygen levels, in 103 Long COVID participants using Cardiopulmonary Exercise Testing (CPET) (43). This study highlighted autonomic dysregulation, characterized by reduced heart rate variability, increased sympathetic activity, and diminished parasympathetic tone, as key contributors to decreased physical capacity and exercise performance (43). These physiological changes underscore the importance of autonomic balance in maintaining cardiovascular efficiency and exercise performance (44). Additionally, CPET-based studies suggests that exercise intolerance in Long COVID is primarily driven by peripheral factors rather than lung or circulatory dysfunction (17), and is associated with lower peak VO2 and persistent dyspnea (45–48).

In exploring the factors contributing to reduced physical performance, we also examined differences within our Long COVID group. Significant differences were identified between hospitalized and non-hospitalized participants in terms of BMI and the level of comorbidity. Consistent with prior studies, 71.4% of hospitalized individuals with Long COVID in our study had a BMI > 30 kg/m^2^. While this highlights a high prevalence of obesity among hospitalized individuals in our cohort, it is important to note that this finding is based on a small sample size of hospitalized individuals (*n* = 14) and does not imply a causal relationship between obesity and hospitalization following COVID-19 infection. Nonetheless, other studies suggest that obesity may increase the likelihood of hospitalization following a COVID-19 infection (9, 49). For instance, a retrospective analysis of 124 intensive care COVID-19 patient found that nearly half had a BMI greater than 30 kg/m^2^ (50). This could be explained by the impact of obesity on immune cells functionality, potentially compromising the body's capacity to mount an efficient response to infections (51). This insight underscores the multifaceted relationship between obesity, immune response, and COVID-19 severity, which is crucial for developing targeted strategies to manage and mitigate the impact of COVID-19, particularly in populations with higher BMI.

Individuals with a higher number of comorbidities are considered more likely to require hospitalization, as evidenced by a study involving 364 participants, of whom 128 were hospitalized (52). Despite the small sample size in our cohort, we observed a similar trend, with hospitalized individuals in the LCG having significantly higher SCQ scores compared to non-hospitalized individuals (5.8 ± 5.2 vs. 3.4 ± 3.1, *p* = 0.02). This finding aligns with a study showing that the presence of multiple comorbidities increased hospitalization rates by 20%–40% (53). Additionally another study reported that 83.7% of patients hospitalized due to COVID-19 had at least one comorbidity (54).

There were no differences in questionnaire scores and physical impairments for participants in the LCG when categorized according to the time since infection. This suggests that functional limitations persist at a stable level even in cases of longstanding infections. Thus, individuals with Long COVID consistently exhibit comparable levels of limitations both subjectively, as reflected in questionnaires scores, and objectively, as gauged by physical tests. Similarly, in a cohort study, Agergaard et al. revealed that 1.5 years after infection, patients still did not experience any clinically meaningful decline in the severity of Long COVID. Moreover, 57% (*n* = 429) of patients showed no improvement 1.5 year after infection (55). Another study showed reduced maximal and submaximal physical performance, along with compromised quality of life, persisting in Long COVID patients 43 weeks post-infection (18).

No statistically significant difference was observed between men and women in terms of self-reported outcomes, including level of comorbidities, HRQoL, sleep quality, pain, and fatigue. This suggests that, based on self-reported measures, men and women with Long COVID exhibit similar subjective experiences. However, given the notably low number of male participants in our sample, these findings should be interpreted with caution, as they may not fully capture potential sex or gender differences reported in other studies. For instance, a study with a sample consisting of 80.6% women and 19.4% men (*n* = 206) found that pain was highly prevalent in both genders, while HRQoL was consistently lower in women (56). Similarly, a systematic review and meta-analysis reported that women with Long COVID are more likely to experience fatigue compared to men (57). Additionally, a study involving 1,297 individuals with Long COVID (51.5% women) revealed that women reported a higher number of symptoms on average compared to men (3.6 vs. 3.1, *p* < 0.001) and exhibited a significantly greater prevalence of comorbidities (58). When comparing physical tests, notable differences were identified between men and women, potentially due to inherent physiological factors. Differences in muscle composition and cardiovascular capacities between the sexes could contribute to these observed disparitiess (59). For example, significant differences were found when the 6MWT was compared between men and women in both the SCG and CG. However, when comparing the groups by sex, both men and women in the LCG exhibited reduced performance compared to their counterparts in the SCG and CG.

Lower HRQoL in individuals with Long COVID was associated to a higher score of fatigue, pain, comorbidity, and lower average daily steps count. These findings align with existing literature, as persistent fatigue is known to result in reduced energy, motivation, and overall well-being, consequently affecting the capacity to engage in physical activities (60). A prospective longitudinal study reported that individuals with Long COVID experienced lower quality of life, particularly in domains related to physical functioning, energy and fatigue, social functioning, pain, and general health (61). Enhanced physical capacity is recognized for its potential to improve quality of life, facilitating active participation in daily activities, promoting independence, and increasing overall satisfaction (62, 63). Conversely, reduced physical capacity can negatively affect quality of life by limiting engagement in diverse activities (62, 63). In line with these observations, a recent study involving 230 individuals with Long COVID reported a high prevalence of fatigue (94.5%) and associated reductions in quality of life (64). Another two-year longitudinal study found that Long COVID was significantly associated with reduced quality of life across multiple domains, with symptoms peaking six months after the initial infection and often resulting in activity limitations (65). These findings underscore the profound and multifaceted impact of Long COVID on quality of life, particularly through its effects fatigue, physical capacity, and activity limitations. Addressing these challenges requires a comprehensive approach that integrates physical rehabilitation, symptom management, and tailored interventions to mitigate the long-term consequences of Long COVID and improve overall well-being.

## Conclusion

Individuals with Long COVID had lower physical capacities than those who experienced short COVID or no COVID, characterized by higher levels of fatigue, pain, comorbidity, and lower HRQoL. Moreover, our research has identified several factors explaining HRQoL in Long COVID participants. Specifically, a negative association was observed with the levels of pain, comorbidity, and fatigue, while a positive association was found with average daily steps count.

## Data Availability

Supplementary analyses categorizing the Long COVID group by sex, hospitalization status, comorbidities, and time since infection are provided in the Supplementary Material. Further inquiries can be directed to the corresponding author.
