# Supplementary material for: Physical impairments in individuals with Long COVID
Source: Front Sports Act Living. 2025 Jan 30;7:1511942. doi: 10.3389/fspor.2025.1511942 (PMC11821922; doi:10.3389/fspor.2025.1511942)
Supplement: Supplementary file 1 [file Table1.docx]

**Supplementary File 1. Long COVID results categorized by sex**

|  | **Woman**  **(n= 102)** | **Men**  **(n=18)** | ***p* value** |
| --- | --- | --- | --- |
| Age, years;  mean (SD) | 43.5 (10.9) | 47.9 (12.5) | 0.1 |
| BMI; mean (SD) | 27.2 (6.2) | 29.6 (5.7) | 0.1 |
| Time since first infection; mean (SD) | 334 (146.0) | 306 (147.0) | 0.5 |
| **Smoking status; n (%)** | | |  |
| Former smoker | 22 (21.6) | 4 (22.2) | 0.4 |
| Never smoked | 74 (72.5) | 12 (66.7) |  |
| Active smoker | 4 (3.9) | 2 (11.1) |  |
| Missing data | 2 (2.0) | 0 (0.0) |  |
| **Self-Reported Questionnaires; mean (SD)** | | | |
| EQ-5D-5L | 0.8 (0.1) | 0.8 (0.1) | 0.9 |
| PSQI | 9.5 (4.5) | 9.5 (4.5) | 0.9 |
| BPI-Pain Severity | 3.3 (2.0) | 2.6 (1.9) | 0.2 |
| BPI-Pain Interference | 3.5 (2.5) | 3.3 (2.6) | 0.9 |
| SCQ | 4.2 (3.8) | 4.1 (3.4) | 0.9 |
| FSS | 47.3 (11.9) | 43.3 (12.0) | 0.2 |
| **Objective Clinical Tests; mean (SD)** | | | |
| HGS | 28.9 (7.1) | 47.1 (9.7) | **<.001** |
| SPPB | 10.5 (2.0) | 11.1 (1.2) | 0.2 |
| 6MWT | 454 (134) | 528 (81.6) | **0.04** |
| Highest perceived exertion during 6MWT | 4.0 (1.7) | 3.2 (1.5) | 0.1 |
| **Physical Activity (Fitness Tracker Watch); mean (SD)** | | |  |
| Highest HR | 138 (20.7) | 139 (12.1) | 0.8 |
| HR in rest | 66.6 (6.5) | 66.1 (5.8) | 0.8 |
| Intensive minutes | 18.4 (19.2) | 16.7 (20.6) | 0.8 |
| Step count | 5326 (2397) | 5994 (2324) | 0.4 |

BMI: Body Mass Index; EQ-5D-5L: EuroQol Group's 5-Dimension 5-Level Questionnaire; PSQI: Pittsburgh Sleep Quality Index; SCQ: Self-Administered Comorbidity Questionnaire; BPI-SF: Brief Pain Inventory - Short Form; FSS: Fatigue Severity Scale; HGS: Hand Grip Strength; SPPB: Short Physical Performance Battery; 6MWT: 6-Minute Walk Test; HR: Heart Rate.

**Supplementary File 2. Long COVID results categorized by hospitalization**

|  | **Hospitalized  (n=14)** | **Non-Hospitalized  (n=106)** | ***p* value** |
| --- | --- | --- | --- |
| Age, years; mean (SD) | 48.8 (13.7) | 43.6 (10.8) | 0.1 |
| BMI; mean (SD) | 33.7 (6.7) | 26.7 (5.6) | **<.001*** |
| Women; n (%) | 9 (64.2) | 93 (87.7) | **0.02** |
| Time since first infection; mean (SD) | 327.6 (166.1) | 329.9 (143.4) | 0.9 |
| **Smoking status; n (%)** | | |  |
| Former smoker | 3 (21.4) | 23 (21.7) | 0.6 |
| Never smoked | 11 (78.6) | 75 (70.8) |  |
| Active smoker | 0 (0.0) | 6 (5.7) |  |
| Missing data | 0 (0.0) | 2 (1.9) |  |
| **Self-Reported Questionnaires; mean (SD)** | | | |
| EQ-5D-5L | 0.8 (0.1) | 0.8 (0.1) | 0.5 |
| PSQI | 9.3 (5.1) | 9.5 (4.4) | 0.8 |
| BPI-Pain Severity | 3.2 (2.0) | 2.7 (1.9) | 0.4 |
| BPI-Pain Interference | 3.5 (2.5) | 2.9 (2.5) | 0.4 |
| SCQ | 5.8 (5.2) | 3.4 (3.1) | **0.02** |
| FSS | 48 (14.4) | 48.0 (11.7) | 0.7 |
| **Objective Clinical Tests; mean (SD)** | | | |
| HGS | 39.4 (15.4) | 30.4 (8.5) | **0.001** |
| SPPB | 10.1 (2.0) | 10.6 (1.9) | 0.4 |
| 6MWT | 437.5 (103.6) | 467.8 (133.5) | 0.4 |
| Highest perceived exertion during 6MWT | 3.9 (1.7) | 3.9 (1.7) | 0.9 |
| **Physical Activity (Fitness Tracker Watch); mean (SD)** | | |  |
| Highest HR | 138 (10.2) | 138 (20.8) | 0.9 |
| HR in rest | 69.3 (6.5) | 66.1 (6.4) | 0.1 |
| Intensive minutes | 14.0 (20.0) | 18.8 (19.4) | 0.4 |
| Step count | 4467 (3164) | 5527 (2287) | 0.2 |

BMI: Body Mass Index; EQ-5D-5L: EuroQol Group's 5-Dimension 5-Level Questionnaire; PSQI: Pittsburgh Sleep Quality Index; SCQ: Self-Administered Comorbidity Questionnaire; BPI-SF: Brief Pain Inventory - Short Form; FSS: Fatigue Severity Scale; HGS: Hand Grip Strength; SPPB: Short Physical Performance Battery; 6MWT: 6-Minute Walk Test; HR: Heart Rate.

**Supplementary File 3. Long COVID results categorized by number of comorbidities**

|  | **0 comorbidity**  **(n= 28)** | **1-2 comorbidity**  **(n= 65)** | **>3 comorbidity**  **(n=27)** | ***p* value** |
| --- | --- | --- | --- | --- |
| Age, years; mean (SD) | 39.6 (10.6) | 43.9 (11.0) | 49 (10.5) | **0.004** |
| BMI; mean (SD) | 25.6 (4.5) | 28.0 (6.8) | 28.6 (5.9) | 0.2 |
| Women; n (%) | 24 (85.7) | 55 (84.6) | 23 (85.2) | 0.99 |
| Time since first infection; mean (SD) | 279.9 (116.2) | 321.3 (147.4) | 401.2 (145.7) | **0.006** |
| **Smoking status; n (%)** | | | |  |
| Former smoker | 7 (25.0) | 13 (20.0) | 6 (22.2) | 0.7 |
| Never smoked | 21(75.0) | 48 (73.8) | 17 (63.0) |  |
| Active smoker | 0 (0.0) | 4 (6.2) | 2 (7.4) |  |
| Missing data | 0 (0.0) | 0 (0.0) | 2 (7.4) |  |
| **Self-Reported Questionnaires; mean (SD)** | | | |  |
| EQ-5D-5L | 0.9 (0.1) | 0.8 (0.1) | 0.7 (0.1) | **<.001 *** |
| PSQI | 9.7 (4.3) | 9.2 (4.3) | 10.0 (5.2) | 0.7 |
| BPI-Pain Severity | 2.4 (2.1) | 3.1 (1.8) | 4.2 (1.8) | **0.002** |
| BPI-Pain Interference | 2.6 (2.5) | 3.3 (2.5) | 4.5 (2.3) | **0.02** |
| SCQ | 0.5 (1.4) | 3.7 (1.8) | 9.2 (3.5) | **<.001 *** |
| FSS | 46.1 (13.3) | 45.5 (12.5) | 50.5 (8.4) | 0.2 |
| **Objective Clinical Tests; mean (SD)** | | | |  |
| HGS | 33.7 (8.8) | 32.1 (10.0) | 27.8 (10.0) | 0.07 |
| SPPB | 10.8 (1.8) | 10.7 (1.9) | 10.0 (1.9) | 0.3 |
| 6MWT | 494 (150) | 474 (123) | 413 (116) | **0.049** |
| Highest perceived exertion during 6MWT | 3.9 (1.6) | 3.6 (1.7) | 4.3 (1.8) | 0.3 |
| **Physical Activity (Fitness Tracker Watch); mean (SD)** | | | | |
| Highest HR | 136 (15.8) | 139 (23.5) | 136 (12.4) | 0.8 |
| HR in rest | 67.4 (6.4) | 65.7 (6.7) | 67.7 (5.7) | 0.3 |
| Intensive minutes | 26.3 (23.4) | 17.1 (17.0) | 12.5 (18.4) | **0.04** |
| Step count | 6337 (2620) | 5303 (2078) | 4708 (2665) | 0.06 |

BMI: Body Mass Index; EQ-5D-5L: EuroQol Group's 5-Dimension 5-Level Questionnaire; PSQI: Pittsburgh Sleep Quality Index; SCQ: Self-Administered Comorbidity Questionnaire; BPI-SF: Brief Pain Inventory - Short Form; FSS: Fatigue Severity Scale; HGS: Hand Grip Strength; SPPB: Short Physical Performance Battery; 6MWT: 6-Minute Walk Test; HR: Heart Rate.

**Supplementary File 4. Long COVID results categorized by time since infection**

|  | **<6 months (n=21)** | **6 and 12 months**  **(n=52)** | **>12 months (n=47)** | ***P* value** |
| --- | --- | --- | --- | --- |
| Age, years; mean (SD) | 39.5 (10.2) | 43.9 (9.8) | 46.5 (12.6) | 0.06 |
| BMI; mean (SD) | 25.7(5.4) | 27 (4.7) | 29.0 (7.6) | 0.1 |
| Women; n (%) | 18 (85.7) | 43 (82.7) | 41 (87.2) | 0.8 |
| Time since first infection; mean (SD) | 132.7 (30.3) | 276.5 (54.7) | 476.4 (90.5) | **<.001** |
| **Smoking status; n (%)** | | | |  |
| Former smoker | 4 (19.0) | 11 (21.2) | 11 (23.4) | 0.7 |
| Never smoked | 17 (81.0) | 35 (67.3) | 34 (72.3) |  |
| Active smoker n (%) | 0 (0.0) | 4 (7.7) | 2 (4.3) |  |
| Missing data | 0 (0.0) | 2 (3.8) | 0 (0.0) |  |
| **Self-Reported Questionnaires; mean (SD)** | | | |  |
| EQ-5D-5L | 0.8 (0.1) | 0.8 (0.1) | 0.8 (0.1) | 0.2 |
| PSQI | 10.9 (4.6) | 9.3 (4.3) | 9.1 (4.7) | 0.3 |
| BPI-Pain Severity | 2.6 (2.3) | 3.2 (2.0) | 3.3 (1.9) | 0.4 |
| BPI-Pain Interference | 3.2 (2.6) | 3.6 (2.5) | 3.2 (2.5) | 0.7 |
| SCQ | 3.9 (3.3) | 3.5 (3.3) | 5.1 (4.2) | 0.09 |
| FSS | 51.3 (6.9) | 43.7 (13.7) | 48.0 (10.9) | **0.03** |
| **Objective Clinical Tests; mean (SD)** | | | |  |
| HGS | 32.1 (9.7) | 33.2 (9.3) | 29.3 (10.4) | 0.1 |
| SPPB | 10.6 (2.8) | 10.6 (1.6) | 10.5 (1.7) | 0.9 |
| 6MWT | 496 (115) | 484 (120) | 428 (142) | 0.05 |
| Highest perceived exertion during 6MWT | 3.9 (1.6) | 3.9 (1.8) | 3.8 (1.7) | 0.9 |
| **Physical Activity (fitness tracker watch); mean (SD)** | | |  |  |
| Highest HR | 134 (12.3) | 137 (10.9) | 140 (28.1) | 0.5 |
| HR in rest | 64.1 (6.1) | 66.6 (6.3) | 67.4 (6.6) | 0.2 |
| Intensive minutes | 11.6 (12.6) | 20.6 (20.2) | 18.5 (20.5) | 0.3 |
| Step count | 5669 (2196) | 5879 (2099) | 4794 (2659) | 0.1 |

BMI: Body Mass Index; EQ-5D-5L: EuroQol Group's 5-Dimension 5-Level Questionnaire; PSQI: Pittsburgh Sleep Quality Index; SCQ: Self-Administered Comorbidity Questionnaire; BPI-SF: Brief Pain Inventory - Short Form; FSS: Fatigue Severity Scale; HGS: Hand Grip Strength; SPPB: Short Physical Performance Battery; 6MWT: 6-Minute Walk Test; HR: Heart Rate.
